# Effect of lysergic acid diethylamide (LSD) on reinforcement learning in humans

**DOI:** 10.1017/S0033291722002963

**Published:** 2023-10

**Authors:** Jonathan W. Kanen, Qiang Luo, Mojtaba Rostami Kandroodi, Rudolf N. Cardinal, Trevor W. Robbins, David J. Nutt, Robin L. Carhart-Harris, Hanneke E. M. den Ouden

**Affiliations:** 1 Department of Psychology, University of Cambridge, Cambridge, UK; 2 Behavioural and Clinical Neuroscience Institute, University of Cambridge, Cambridge, UK; 3 National Clinical Research Center for Aging and Medicine at Huashan Hospital, State Key Laboratory of Medical Neurobiology and Ministry of Education Frontiers Center for Brain Science, Institutes of Brain Science and Institute of Science and Technology for Brain-Inspired Intelligence, Fudan University, Shanghai, 200433, China; 4 Center for Computational Psychiatry, Ministry of Education-Key Laboratory of Computational Neuroscience and Brain-Inspired Intelligence, Human Phenome Institute, Fudan University, Shanghai, 200032, China; 5 Shanghai Key Laboratory of Mental Health and Psychological Crisis Intervention, School of Psychology and Cognitive Science, East China Normal University, Shanghai, 200241, China; 6 Department of Cognitive Science and Artificial Intelligence, Tilburg University, Tilburg, The Netherlands; 7 Donders Institute for Brain, Cognition and Behaviour, Radboud University, Nijmegen, The Netherlands; 8 Department of Psychiatry, University of Cambridge, Cambridge, UK; 9 Cambridgeshire and Peterborough NHS Foundation Trust, Cambridge, UK; 10 Department of Brain Sciences, Centre for Psychedelic Research, Imperial College London, London, UK; 11 Neuroscape Psychedelics Division, University of California San Francisco, San Francisco, California, USA

**Keywords:** 5-HT2A, cognitive flexibility, computational modeling, LSD, probabilistic reversal learning, psychedelics, reinforcement learning, serotonin

## Abstract

**Background:**

The non-selective serotonin 2A (5-HT_2A_) receptor agonist lysergic acid diethylamide (LSD) holds promise as a treatment for some psychiatric disorders. Psychedelic drugs such as LSD have been suggested to have therapeutic actions through their effects on learning. The behavioural effects of LSD in humans, however, remain incompletely understood. Here we examined how LSD affects probabilistic reversal learning (PRL) in healthy humans.

**Methods:**

Healthy volunteers received intravenous LSD (75 *μ*g in 10 mL saline) or placebo (10 mL saline) in a within-subjects design and completed a PRL task. Participants had to learn through trial and error which of three stimuli was rewarded most of the time, and these contingencies switched in a reversal phase. Computational models of reinforcement learning (RL) were fitted to the behavioural data to assess how LSD affected the updating (‘learning rates’) and deployment of value representations (‘reinforcement sensitivity’) during choice, as well as ‘stimulus stickiness’ (choice repetition irrespective of reinforcement history).

**Results:**

Raw data measures assessing sensitivity to immediate feedback (‘win-stay’ and ‘lose-shift’ probabilities) were unaffected, whereas LSD increased the impact of the strength of initial learning on perseveration. Computational modelling revealed that the most pronounced effect of LSD was the enhancement of the reward learning rate. The punishment learning rate was also elevated. Stimulus stickiness was decreased by LSD, reflecting heightened exploration. Reinforcement sensitivity differed by phase.

**Conclusions:**

Increased RL rates suggest LSD induced a state of heightened plasticity. These results indicate a potential mechanism through which revision of maladaptive associations could occur in the clinical application of LSD.

## Introduction

Research into lysergic acid diethylamide (LSD) as a potential therapeutic agent in psychiatry has been revitalised in recent years (Nutt & Carhart-Harris, 2020; Vollenweider & Preller, 2020). Theories on the putative beneficial effects of LSD on mental health centre on its effects on learning and plasticity (Carhart-Harris & Nutt, 2017), yet a limited number of human studies have examined its effect on instrumental learning and behavioural or cognitive flexibility (Hutten et al., 2020; Pokorny, Duerler, Seifritz, Vollenweider, & Preller, 2019). LSD acts principally but not exclusively as an agonist at the serotonin (5-HT; 5-hydroxytryptamine) 2A (5-HT_2A_) receptor (Marona-Lewicka & Nichols, 2007; Marona-Lewicka, Thisted, & Nichols, 2005; Nichols, 2016). Indeed, blocking 5-HT_2A_ receptors inhibits the psychedelic effects of LSD (Nichols, 2016). The 5-HT_2A_ receptor is involved in plasticity (Barre et al., 2016; Vaidya, Marek, Aghajanian, & Duman, 1997) and its modulation represents a putative neurobiological mechanism through which LSD could facilitate the revision of maladaptive associations (Carhart-Harris & Nutt, 2017). Indeed, LSD and 5-HT_2A_ agonists have been shown to improve associative learning in non-human animals (Harvey, 2003; Harvey, Gormezano, Cool-Hauser, & Schindler, 1988; Romano et al., 2010; Schindler, Gormezano, & Harvey, 1986).

Serotonin is critically involved in adapting behaviour flexibly as environmental circumstances change (Barlow et al., 2015; Brigman et al., 2010; Clarke, Dalley, Crofts, Robbins, & Roberts, 2004; Furr, Danet Lapiz-Bluhm, & Morilak, 2012; Matias, Lottem, Dugué, & Mainen, 2017; Lapiz-Bluhm et al., 2009; Rygula et al., 2015), as well as processing aversive outcomes (Bari et al., 2010; Chamberlain et al., 2006; Cools, Roberts, & Robbins, 2008; Crockett, Clark, & Robbins, 2009; Dayan & Huys, 2009; Deakin, 2013; den Ouden et al., 2013; Geurts, Huys, den Ouden, & Cools, 2013). Both can be modelled in a laboratory setting using PRL paradigms. In these, individuals learn by trial and error the most adaptive action, in an ‘acquisition’ stage, and this rule eventually changes in a ‘reversal’ phase (Lawrence, Sahakian, Rogers, Hodges, & Robbins, 1999). Profound neurotoxin-induced depletion of serotonin from the marmoset orbitofrontal cortex (OFC) causes perseverative, stimulus-bound behaviour (Walker, Robbins, & Roberts, 2009) – an impaired ability to update action upon reversal (Clarke et al., 2004). At the same time, repeated dosing of a selective serotonin reuptake inhibitor (SSRI) improved reversal learning in rats (Bari et al., 2010). Acute administration of SSRIs, meanwhile, has resulted in an increased sensitivity to negative feedback (referred to as ‘lose-shift’ behaviour) in healthy humans (Chamberlain et al., 2006; Skandali et al., 2018) and rats (Bari et al., 2010). Indeed, the latter effect may be attributed to findings that acute SSRI administration can paradoxically lower serotonin concentration in projection areas in monkeys and healthy humans (Nord, Finnema, Halldin, & Farde, 2013), highlighting the complexity of some serotonergic effects.

More specifically, a number of studies have implicated 5-HT_2A_ receptor function in reversal learning. Furr et al. (2012) showed 5-HT_2A_ receptors in the rat OFC contributed to improved reversal learning following chronic SSRI administration. Barlow et al. (2015) reported that highly perseverative rats during reversal learning had reduced 5-HT_2A_ receptors in the OFC. Boulougouris, Glennon, and Robbins (2008) demonstrated that systemic 5-HT_2A_ antagonism impaired reversal learning in rats. At the same time, antagonism of 5-HT_2A_ in the mouse OFC enhanced perseveration during reversal learning whereas 5-HT_2A_ antagonism in the dorsomedial striatum improved reversal learning (Amodeo, Rivera, Cook, Sweeney, & Ragozzino, 2017). These anatomical functional differences may inform the reconciliation of other rodent studies on 5-HT_2A_ and reversal learning that have employed systemic drug administration (Amodeo, Jones, Sweeney, & Ragozzino, 2014, 2020; Baker, Thompson, Sweeney, & Ragozzino, 2011; Odland, Kristensen, & Andreasen, 2021).

In addition to affecting the serotonin system, LSD has dopamine type 2 (D_2_) receptor agonist properties (Marona-Lewicka et al., 2005; Marona-Lewicka & Nichols, 2007; Nichols, 2004). Dopamine is particularly well known to play a fundamental role in learning from feedback (Schultz, 2019; Schultz, Dayan, & Montague, 1997) putatively mediating plasticity changes during associative learning (Shen, Flajolet, Greengard, & Surmeier, 2008; Yin & Knowlton, 2006). Meanwhile, dopamine depletion of the marmoset caudate nucleus, like serotonergic OFC depletion, also induced perseveration (Clarke, Hill, Robbins, & Roberts, 2011). Additionally, there is a body of evidence, across species, that D_2_-modulating agents affect instrumental reversal learning (Boulougouris, Castañé, & Robbins, 2009; Kanen, Ersche, Fineberg, Robbins, & Cardinal, 2019; Lee, Groman, London, & Jentsch, 2007).

Human studies of LSD have employed a variety of behavioural measures including facial emotion recognition, empathy, and social behaviour (Dolder, Schmid, Müller, Borgwardt, & Liechti, 2016); response inhibition (Schmidt et al., 2018); prepulse inhibition (Schmid *et al*. 2015); working memory and risk-based decision-making (Family et al., 2020; Pokorny et al., 2019); processing social influence (Duerler, Schilbach, Stämpfli, Vollenweider, & Preller, 2020); semantic processing (Family et al., 2016); attention, information processing, and cognitive control (Family et al., 2020; Hutten et al., 2020); time perception (Yanakieva et al., 2019); paired associates learning and memory, balance, and proprioception (Family et al., 2020). The effects of psilocybin and 3,4-methylenedioxymethamphetamine (MDMA), which are also non-selective 5-HT_2A_ agonists, in humans have also been studied in relation to episodic memory (Barrett, Carbonaro, Hurwitz, Johnson, & Griffiths, 2018; Doss, Weafer, Gallo, & De Wit, 2018).

Higher-order cognitive flexibility, on a set-shifting task, was impaired by acute intoxication with LSD in healthy humans (Pokorny et al., 2019). Meanwhile, psilocybin increased higher-order cognitive flexibility (set shifting), subsequent to drug treatment, in individuals with major depressive disorder (Doss et al., 2021). Ayahuasca, another psychedelic non-selective 5-HT_2A_ agonist, and psilocybin have been shown to increase creative thinking during and after drug administration, which was interpreted as increased psychological flexibility (Kuypers et al., 2016; Mason, Mischler, Uthaug, & Kuypers, 2019). Meanwhile, healthy human behaviour on an outcome devaluation task, used to parse habitual *v.* goal-directed action, was not impaired by LSD (Hutten et al., 2020).

Here, we studied healthy human volunteers to examine the effects of LSD on a widely used translational measure of instrumental conditioning and behavioural/cognitive flexibility: probabilistic reversal learning (PRL). In contrast to the set-shifting and outcome devaluation tasks used previously, PRL models fundamental aspects of choice behaviour under uncertainty (probabilistic reinforcement) and when flexibility is required. We explored how LSD altered not only overt choice behaviour during PRL (using classical statistics) but also the underlying learning mechanisms, using computational models of reinforcement learning (RL, using Bayesian statistics), which have not been employed in previous studies. Utilising PRL in a placebo-controlled study of healthy human volunteers, the aim of the current experiment was to inform the psychological mechanisms by which LSD could have salubrious effects on mental health.

Based on raw data measures, we predicted LSD would modulate either sensitivity to negative feedback or the impact of learned values on subsequent perseverative behaviour (den Ouden et al., 2013). Measuring ‘staying’ (repeating a choice) or ‘shifting’ (choosing another stimulus) after wins or losses assesses sensitivity to immediate reinforcement but does not account for the integration of feedback history across multiple experiences to influence behaviour (Daw, 2011). To this end, we applied computational models of RL. The expected value of choice options, for example, increases or decreases dynamically based on reward or punishment prediction errors (experienced better or worse than expected outcomes). A key objective of this study was to evaluate the effects of LSD on the rate at which value is updated (‘learning rates’) – in essence, does LSD affect how quickly expectations change following reinforcement? Another question of interest was whether LSD modulates exploratory behaviour. We tested two varieties of exploration. First, we addressed whether LSD impacts the extent to which behaviour is guided by exploiting the more highly valued choice or, conversely, an exploratory pattern that is less guided by value (termed high or low ‘reinforcement sensitivity,’ respectively). The second variety of exploration (low ‘stimulus stickiness’) was value-free rather than value-based in that it represents a tendency to explore (rather than repeat) different choices (stimuli) to what has been chosen previously, regardless of the action's outcome (irrespective of value representations).

## Materials and methods

### Subjects and drug administration

Nineteen healthy volunteers (mean age 30.6; 15 males), over the age of 21, attended two sessions at least two weeks apart where they received either intravenous LSD (75 *μ*g in 10 mL saline) or placebo (10 mL saline), in a single-blind within-subjects balanced-order design. Whereas 20 participants were included in the original study (Carhart-Harris et al., 2016b), one participant did not complete the PRL task; therefore, 19 participants are reported here. Demographic information is provided in online Supplementary Table S1. All participants provided written informed consent after briefing on the study and screening. Participants had no personal history of diagnosed psychiatric disorder, or immediate family history of a psychotic disorder. Other inclusion criteria were a normal electrocardiogram (ECG), normal screening blood tests, negative urine tests for pregnancy and recent recreational drug use, a negative breathalyser test for recent alcohol use, alcohol use limited to less than 40 UK units per week, and absence of a significant medical condition. Participants had previous experience with a classic psychedelic drug [e.g. LSD, mescaline, psilocybin/magic mushrooms, or dimethyltryptamine (DMT)/ayahuasca] without an adverse reaction, and had not used these within six weeks of the study. Screening was conducted at the Imperial College London Clinical Research Facility (ICRF) at the Hammersmith Hospital campus, and the study was carried out at the Cardiff University Brain Research Imaging Centre (CUBRIC). Participants were blinded to the condition but the experimenters were not. A cannula was inserted and secured in the antecubital fossa and injection was performed over the course of two minutes. Participants reported noticing subjective effects of LSD five to 15 min after dosing. The PRL task was administered approximately five hours after injection. Once the subjective drug effects subsided, a psychiatrist assessed suitability for discharge. This experiment was part of a larger study, the data from which are published elsewhere (e.g. Carhart-Harris *et al*. 2016b). Additional information can be found in Carhart-Harris *et al*. (2016b).

### Probabilistic reversal learning task

**Fig. 1.**
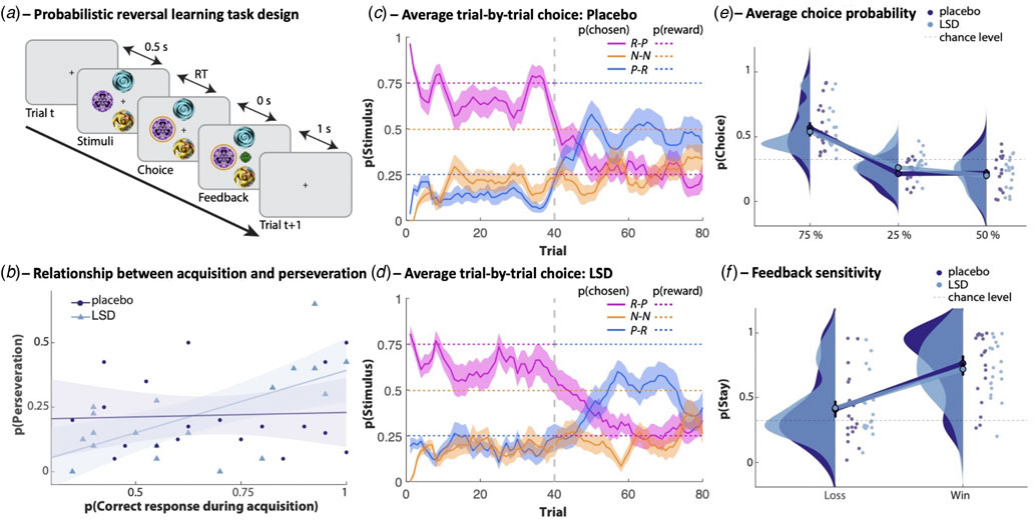
(a) Schematic of the PRL task. Subjects chose one of three stimuli. The timeline of a trial is depicted: stimuli appear, a choice is made, the outcome is shown, a fixation cross is presented during the intertrial interval, stimuli appear for the next trial (etc.) (RT, reaction time). One stimulus delivered positive feedback (green smiling face) with a 75% probability, one with 50%, and one with 25%. The probabilistic alternative was negative feedback (red sad face). Midway through the task, the contingencies for the best and worst stimuli swapped. s, seconds. (b) Better initial learning was predictive of more perseveration on LSD and not on placebo. Shading indicates ± 1 standard error of the mean (s.e.). (c) Trial-by-trial average probability of choosing each stimulus, averaged over subjects during the placebo session. A sliding 5-trial window was used for smoothing. The vertical dotted line indicates the reversal of contingencies. R-P indicates mostly rewarded stimulus, later mostly punished. N-N indicates neutral stimulus during both acquisition and reversal. P-R indicates mostly punished stimulus, later mostly rewarded stimulus. Shading indicates ± 1 s.e. (d) Trial-by-trial average probability of choosing each stimulus, averaged over subjects during the LSD session. A sliding 5-trial window was used for smoothing. The vertical dotted line indicates the reversal of contingencies. R-P indicates mostly rewarded stimulus, later mostly punished. N-N indicates neutral stimulus during both acquisition and reversal. P-R indicates mostly punished stimulus, later mostly rewarded stimulus. Shading indicates ± 1 s.e. (e) Distributions depicting the average per-subject probability (scattered dots) of choosing each stimulus while under placebo (shown in dark blue) and LSD (light blue). The mean value for each distribution is illustrated with a single dot at the base of each distribution, and the mean values for the probability of choosing different stimuli in each condition are connected by a line. Black error bars around the mean value show ± 1 s.e. Horizontal dotted line indicates chance-level ‘stay’ behaviour (33%). The global probability of choosing each stimulus did not differ between the placebo and LSD conditions. (f) Raw data measures of feedback sensitivity were unaffected by LSD. Distributions depicting the average per-subject probability (scattered dots) of repeating a choice (staying) after receiving positive or negative feedback under placebo (dark blue) and LSD (light blue). The horizontal dotted line indicates chance-level ‘stay’ behaviour (33%).

A schematic of the task is shown in Fig. 1a. On every trial, participants could choose from three visual stimuli, presented at three of four randomised locations on a computer screen. In the first half of the task (40 trials), choosing one of the stimuli resulted in positive feedback in the form of a green smiling face on 75% of trials. A second stimulus resulted in positive feedback 50% of the time, whilst the third stimulus yielded positive feedback on only 25% of trials. Negative feedback was provided in the form of a red frowning face. The first stimulus selected was defined as the initially rewarded stimulus; the choice on trial 1 always resulted in reward. The second stimulus that was selected was defined as the mostly punished stimulus, and by definition the third stimulus was then the ‘neutral’ stimulus. After 40 trials, the most and least optimal stimuli reversed, such that the stimulus that initially was correct 75% of the time was then only correct 25% of the time, and likewise the 25% correct stimulus then resulted in positive feedback on 75% of trials. There were 40 trials in the reversal phase. This is a recently developed version (Rostami Kandroodi et al., 2021) of a widely used PRL task (den Ouden et al., 2013; Lawrence et al., 1999) – novel due to the addition of a 50% ‘neutral’ stimulus in order to distinguish learning to select the mostly rewarding stimulus from learning to avoid the mostly punishing stimulus.

### Raw data measures of behaviour

We examined whether LSD impaired participants' basic overall ability to perform the task by analysing the number of responses made to each stimulus during the acquisition and reversal phases. We measured feedback sensitivity by determining whether participants stayed with the same choice following positive or negative feedback (win-stay or lose-stay). The win-stay probability was defined as the number of times an individual repeated a choice after a win, divided by the number of trials on which positive feedback occurred (opportunities to stay after a win). Lose-stay probability was calculated in the same manner: the number of times a choice was repeated following a loss, divided by the total losses experienced. Note that in previous studies with a choice between only two stimuli (or responses), this metric is usually referred to as ‘win-stay/lose-shift’, which also captures the tendency to repeat (rather than switch) responses following a win, and the tendency to switch (rather than repeat) choices following a loss. Random choice would result in 50% win-stay and 50% lose-shift; however, in the current paradigm with 3 stimuli, this base rate is 33% (win-)stay and 67% (lose-)shift. We therefore encode both variables with respect to the stay (rather than shift) rate, but they are still conceptually identical to earlier studies. Perseveration was defined according to den Ouden et al. (2013) and was assessed based on responses in the reversal phase. A perseverative error occurred when two or more (now incorrect) responses were made to the previously correct stimulus, and these errors could occur at any point in the reversal phase. The first trial in the reversal phase (trial 41 of 80) was excluded from the perseveration analysis, however, as at that point behaviour cannot yet have been shaped by the new feedback structure. Note again that this metric is not entirely identical to the previous studies cited employing two stimuli, as the base-rate choice for each stimulus is now 1/3, so the ‘chance’ level of perseverative errors is lower. Null hypothesis significance tests used *α* = 0.05.

### Computational modelling of behaviour

#### Model fitting, comparison, and interpretation

These methods are based on our previous work (Kanen et al., 2019). We fitted three RL models to the behavioural data using a hierarchical Bayesian method, via Hamiltonian Markov chain Monte Carlo sampling implemented in Stan 2.17.2 (Carpenter et al., 2017). Convergence was checked according to 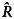, the potential scale reduction factor measure (Brooks & Gelman, 1998; Gelman, Hill, & Yajima, 2012), which approaches 1 for perfect convergence. Values below 1.2 are typically used as a guideline for determining model convergence (Brooks and Gelman 1998). We assumed the three models had the same prior probability (0.33). Models were compared via a bridge sampling estimate of the marginal likelihood (Gronau et al., 2017a), using the ‘bridgesampling’ package in R (Gronau, Singmann, & Wagenmakers, 2017b). Bridge sampling directly estimates the marginal likelihood, and therefore the posterior probability of each model given the data (and prior model probabilities), as well as the assumption that the models represent the entire group of those to be considered. Posterior distributions were interpreted using the 95% highest posterior density interval (HDI), which is the Bayesian ‘credible interval.’ Parameter recovery for this modelling approach has been confirmed in a previous study (Kanen et al., 2019) and is demonstrated in the online Supplementary material.

The Bayesian hierarchy consisted of ‘drug condition’ at the highest level, and ‘subject’ at the level below. For each parameter, each drug condition (e.g. LSD) had its own mean (with a prior that was the same across conditions, i.e. with priors that were unbiased with respect to LSD *v.* placebo). This was then merged with the intersubject variability (assumed to be normally distributed; mean 0 by definition, standard deviation determined by a further prior). The priors used for each parameter are shown in Table 1. For instance, the learning rate for a given subject under LSD was taken as: the group mean LSD value for learning rate, plus the subject-specific component of learning rate. The learning rate for a given subject under placebo was taken as: the group mean placebo value for learning rate, plus the subject-specific component of the learning rate for the same subject. This method accounts for the within-subjects structure of the study design. This was done similarly (and separately) for all other model parameters.

**Table 1.** Prior distributions for model parameters

Model parameters	Models using each parameter	Range	Prior	Reference
Reward learning rate, *α^rew^*	1, 3	[0, 1]	Beta(1.2, 1.2)	den Ouden et al. (2013)
Punishment learning rate, *α^pun^*	1, 3	[0, 1]	Beta(1.2, 1.2)	den Ouden et al. (2013)
Combined reward/punishment learning rate, *α^reinf^*	2	[0, 1]	Beta(1.2, 1.2)	den Ouden et al. (2013)
Reinforcement sensitivity, *τ^reinf^*	1, 2, 3	[0, +∞)	Gamma(*α* = 4.82, *β* = 0.88)	Gershman (2016)
Stimulus stickiness, *τ^stim^*	2, 3	(–∞, +∞)	Normal(0, 1)	Christakou et al. (2013)
Intersubject variability in parameters				
Intersubject standard deviations for *α^rew^*, *α^pun^*, *α^reinf^, τ^stim^*	As above	[0, +∞)	Half-normal: Normal(0, 0.05) constrained to ≥0	Kanen et al. (2019)
Intersubject standard deviations for *τ^reinf^*	As above	[0, +∞)	Half-normal: Normal(0, 1) constrained to ≥0	Kanen et al. (2019)

*rew,* reward; *pun,* punishment; *reinf,* reinforcement; *stim,* stimulus.

To determine the change (LSD – placebo) in parameters, we calculated [group mean LSD learning rate] – [group mean placebo learning rate] for each of the ~8000 simulation runs and tested them against zero via the HDI. This approach also removes distributional assumptions and provides an automatic multiple comparisons correction (Gelman et al., 2012; Gelman & Tuerlinckx, 2000; Kruschke, 2011).

#### Models

The parameters contained in each model are summarised in Tables 1 and 2. With Model 1, we tested the hypothesis that positive *v.* negative feedback guides behaviour differentially, and that LSD affects this. We augmented a basic RL model (Rescorla & Wagner, 1972) with separate learning rates for reward, *α^rew^*, and punishment, *α^pun^*. Positive feedback led to an increase in the value *V_i_* of the stimulus *i* that was chosen, at a speed governed by the *reward learning rate, α^rew^*, via *V_i,t_*_+1_ ← *V_i,t_* + *α^rew^*(*R_t_* – *V_i,t_*). *R_t_* represents the outcome on trial *t* (defined as 1 on trials where positive feedback occurred), and (*R_t_* – *V_i,t_*) the prediction error. On trials where negative feedback occurred, *R_t_* = 0, which led to a decrease in value of *V_i_* at a speed governed by the *punishment learning rate, α^pun^*, according to *V_i,t_*_+1_ ← *V_i,t_* + *α^pun^*(*R_t_* – *V_i,t_*). Stimulus value was incorporated into the final quantity controlling choice according to *Q^reinf^_t_* = *τ^reinf^V_t_*. The additional parameter *τ^reinf^*, termed *reinforcement sensitivity*, governs the degree to which behaviour is driven by reinforcement history. The quantities *Q* associated with the three available choices, for a given trial, were then fed into a standard softmax choice function to compute the probability of each choice:
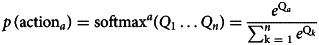
for *n* = 3 choice options. The probability values for each trial emerging from the softmax function (the probability of choosing stimulus 1) were fitted to the subject's actual choices (did the subject choose stimulus 1?). No further softmax inverse temperature was applied (*β* = 1; see below), and as a result the reinforcement sensitivity parameter (*τ^reinf^*) directly represented the weight given to the exponents in the softmax function.

**Table 2.** Model comparison

Rank	Name	Parameters	log marginal likelihood	log posterior *p* (model)
2	Model 1	*α^rew^, α^pun^, τ^reinf^*	−2401.49	−33.28
3	Model 2	*α^reinf^, τ^reinf^, τ^stim^*	−2428.52	−60.32
1	Model 3	*α^rew^, α^pun^, τ^reinf^, τ^stim^*	−2368.21	0

*rew,* reward; *pun,* punishment; *reinf,* reinforcement, *stim,* stimulus; log posterior probabilities are rounded to two decimal places.

Model 2 again augmented a simple RL model, but now also described the tendency to repeat a response, irrespective of the outcome that followed it (in other words, the tendency to ‘stay’ regardless of outcome). With Model 2 we tested the hypothesis that LSD affects this basic perseverative tendency. This was implemented using a ‘stimulus stickiness’ parameter, *τ^stim^*. The stimulus stickiness effect was modelled as *Q^stim^_t_* = *τ^stim^s_t_*_–1_, where *s_t_*_–1_ was 1 for the stimulus that was chosen on the previous trial and was 0 for the other two stimuli. In this model, we used only a single RL rate, *α^reinf^*. Positive reinforcement led to an increase in the value *V_i_* of the stimulus *i* that was chosen, at a speed controlled by the learning rate, *α^reinf^*, via *V_i,t_*_+1_ ← *V_i,t_* + *α^reinf^*(*R_t_* – *V_i,t_*). The final quantity controlling choice incorporated the additional stickiness parameter as *Q_t_* = *Q^reinf^_t_* + *Q^stim^_t_*. Quantities *Q*, corresponding to the three choice options on a given trial, were then fed into the softmax function as above. It should be noted that if *τ^stim^* is not in the model (or is zero), then *τ^reinf^* is mathematically identical to the notion of softmax inverse temperature typically implemented as *β*. The notation *τ^reinf^* is used, however, because it contributes to *Q^reinf^_t_* but not to *Q^stim^_t_*. A standard implementation of *β*, by contrast, would govern the effects of both *Q^reinf^_t_* and *Q^stim^_t_* by weighting the sum of the two (*Q_t_*).

Model 3 was the full model that incorporated separate reward and punishment learning rates as well as the stimulus stickiness parameter. With Model 3, we tested the hypothesis that LSD affects both how positive *v.* negative feedback guides behaviour differentially, and how LSD affects a basic perseverative tendency. Again, the final quantity controlling choice was determined by *Q_t_* = *Q^reinf^_t_* + *Q^stim^_t_*.

## Results

### Learning and perseveration

**Fig. 2.**
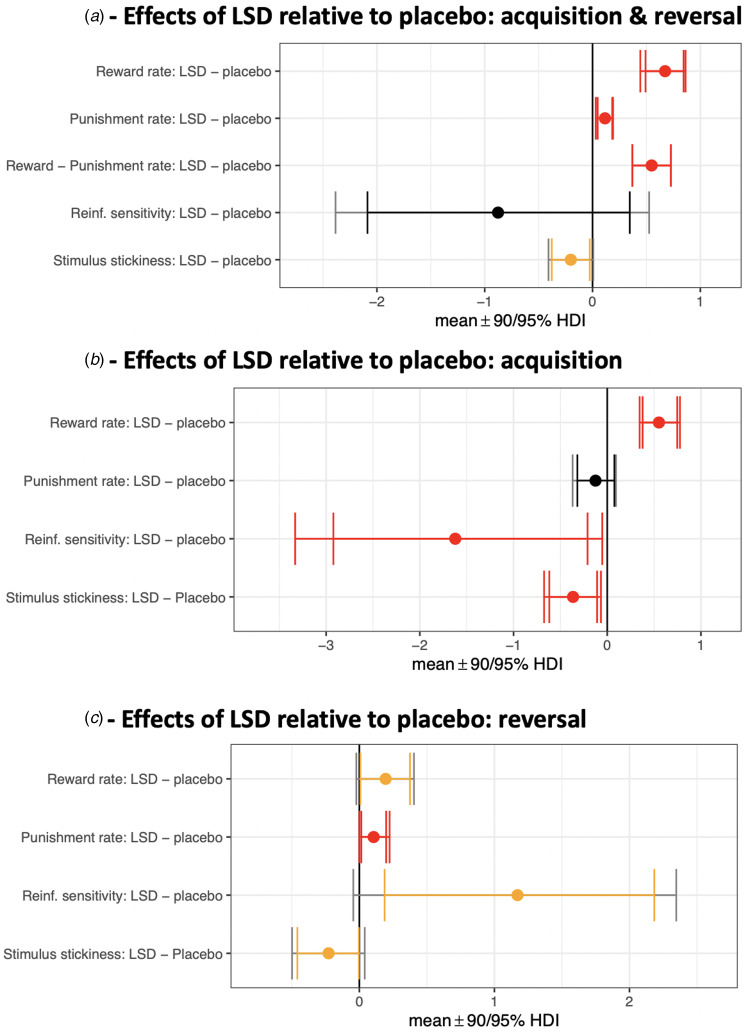
Effects of LSD relative to placebo on model parameters. Contrasts with the posterior 95% (or greater) HDI of the difference between means excluding zero (0 ∉ 95% HDI) are shown in red. Yellow signifies 0 ∉ 90% HDI. (a) Acquisition and reversal phases (all trials) modelled together. The third row represents a difference of differences scores: (*α^rew^*_LSD_ – *α^pun^*_LSD_) – (*α^rew^*_placebo_ – *α^pun^*_placebo_). (b) Isolating the acquisition phase. (c) Isolating the reversal phase.

First, we examined whether LSD altered participants' overall ability to choose the stimulus that led to reward most of the time. Behavioural performance is depicted in Figs 1 and 2. To examine whether LSD affected the number of times each stimulus was chosen, repeated-measures analysis of variance (ANOVA) was conducted with drug (LSD, placebo), phase (acquisition, reversal), and stimulus type (75, 50, or 25% rewarded) as within-subjects factors. This revealed a main effect of stimulus (*F*_1,23_ = 30.66, *p* = 3 × 10^−6^, *η_p_^2^* = 0.63), a stimulus × phase interaction (*F* = 28.62, *p* = 2 × 10^−6^, *η_p_^2^* = 0.61), and no interaction of LSD with stimulus or phase (*F* < 1.5, *p* > 0.24, *η_p_^2^* < 0.08, for terms involving LSD). The number of correct responses did not differ between placebo and LSD during the acquisition (paired-sample *t* test, *t*_18_ = 0.84, *p* = 0.4, *d* = 0.19) or reversal phases (*t*_18_ = 0.23, *p* = 0.8, *d* = 0.05).

We then examined the relationship between initial learning and perseveration, following den Ouden et al. (2013) (Fig. 1b). LSD enhanced the relationship between the number of correct responses during the acquisition phase and the number of perseverative errors made during the subsequent reversal stage [acquisition correct responses (LSD minus placebo) *v.* reversal perseverative errors (LSD minus placebo): linear regression coefficient *β* = 0.56, *p* = 0.002]. Confirming this, making fewer errors during the acquisition phase predicted more perseverative errors when on LSD (*β* = 0.44, *p* = 0.003) but not when under placebo (*β* = 0.04, *p* = 0.8). Perseverative errors, a subset of all reversal errors, alone did not differ between conditions (*t*_18_ = 0.03, *p* = 0.98, *d* = 0.01).

### Feedback sensitivity

We next assessed whether LSD influenced individuals' responses on trials immediately after positive *v.* negative feedback – whether participants stayed with the same choice after a win or a loss (win-stay/lose-stay; Figure 1f). Repeated-measures ANOVA with drug (LSD, placebo) and valence (win, loss) as within-subjects factors revealed a main effect of valence – participants ‘stayed’ more after wins than losses (*F*_1,18_ = 37.76, *p* = 8.0 × 10^–6^, *η*_p_^2^ = 0.68) – and no main effect of LSD (*F*_1,18_ = 0.20, *p* = 0.66, *η_p_^2^* = 0.01). There was also no interaction of valence × LSD (*F*_1,18_ = 0.63, *p* = 0.44, *η_p_^2^* = 0.03).

### Choice of reinforcement learning model

The core modelling results are displayed in Fig. 2. We fitted and compared three RL models. Convergence was good with all three models having 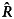 < 1.2. Behaviour was best characterised by a RL model with four parameters (Table 2). The four parameters in the winning model were: (1) reward learning rate, which reflects the degree to which the chosen stimulus value is increased following a positive outcome; (2) punishment learning rate, the degree to which the chosen stimulus value is decreased following a negative outcome; (3) reinforcement sensitivity, the degree to which the values learned through reinforcement contribute to final choice; and (4) ‘stimulus stickiness’, which quantifies the tendency to get ‘stuck’ to a stimulus and choose it because it was chosen on the previous trial, irrespective of the outcome. The last two parameters resemble the explore/exploit trade-off: low values of stickiness or reinforcement sensitivity characterise two different types of exploratory behaviour.

### Reward and punishment learning rates

First, we modelled all 80 trials in the task (both acquisition and reversal phases) and these results are depicted in Fig. 2a. The reward learning rate was significantly elevated on LSD (mean 0.87) compared to placebo (mean 0.28) [with the posterior 99.9% HDI of the difference between these means excluding zero; 0 ∉ 99.9% HDI]. There was also an increased punishment learning rate under LSD (mean 0.48) relative to placebo (mean 0.39) (drug difference, 0 ∉ 99% HDI; Figure 2a 99% HDIs not shown graphically). LSD increased the reward learning rate to a greater extent than the punishment learning rate [(*α^rew,LSD^* – *α^rew,placebo^*) – (*α^pun,LSD^* – *α^pun,placebo^*) > 0; drug difference, 0 ∉ 99% HDI].

To better understand how LSD affected the dynamics of flexible choice behaviour, we then modelled the acquisition and reversal phases separately (40 trials each). During acquisition (Fig. 2b), the reward learning rate was elevated under LSD (mean 0.72) compared to placebo (mean 0.17) (drug difference, 0 ∉ 99% HDI). The punishment learning rate during acquisition, meanwhile, was not significantly elevated under LSD (mean 0.34) compared to placebo (mean 0.47) (no drug difference, 0 ∈ 90% HDI). LSD increased the reward learning rate more than the punishment learning rate [(*α^rew,LSD^* – *α^rew,placebo^*) – (*α^pun,LSD^* – *α^pun,placebo^*) > 0; drug difference, 0 ∉ 99.9% HDI].

During the reversal phase (Fig. 2c), the reward learning rate was elevated under LSD (mean 0.96) compared to placebo (mean 0.77) (drug difference, 0 ∉ 90% HDI) as was the punishment learning rate (LSD mean 0.42; placebo mean 0.31; drug difference, 0 ∉ 90% HDI). During reversal, there was no difference between the effect of LSD on the reward learning rate *v.* on the punishment learning rate [(*α^rew,LSD^* – *α^rew,placebo^*) – (*α^pun,LSD^* – *α^pun,placebo^*) drug difference, 0 ∈ 99.9% HDI].

### Stimulus stickiness and reinforcement sensitivity

Modelling both acquisition and reversal contiguously, stimulus stickiness was lowered by LSD (mean 0.23) relative to placebo (mean 0.43) (drug difference, 0 ∉ 90% HDI; Figure 2a), which is a manifestation of increased exploratory behaviour. Reinforcement sensitivity was not modulated by LSD (LSD mean 4.70, placebo mean 5.57; no drug difference, 0 ∈ 95% HDI). This is in line with the absence of an effect of LSD on the tendency to ‘stay’ following reward or punishment (see analysis of raw data measures above).

When modelling the acquisition phase alone (Fig. 2b), stimulus stickiness was diminished under LSD (mean 0.09) compared to placebo (mean 0.46) (drug difference, 0 ∉ 90% HDI) as was reinforcement sensitivity (LSD mean 4.92; placebo mean 6.54; drug difference, 0 ∉ 90% HDI). In other words, during acquisition, behaviour under LSD was more exploratory as assessed by two metrics – one value-based (reinforcement sensitivity) and one value-free (stimulus stickiness).

When modelling the reversal phase alone (Fig. 2c), stimulus stickiness remained decreased under LSD (mean 0.36) compared to placebo (mean 0.58) (drug difference, 0 ∉ 90% HDI), as during acquisition. Reinforcement sensitivity, however, which had been decreased under LSD during acquisition, was instead increased under LSD during the reversal phase (LSD mean 3.64; placebo mean 2.47; drug difference, 0 ∉ 90% HDI).

### Relationship between model parameters and raw data behavioural measures

Analyses to understand the relationship between computational and raw data measures were conducted. Given the initial finding on the relationship between better acquisition learning and perseveration, the first question addressed was whether the elevated reward learning rate under LSD during acquisition, from the computational model, was predictive of the raw data measure of perseveration from den Ouden et al. (2013). Simple linear regression showed that under LSD, a higher reward learning rate during acquisition predicted significantly more perseverative errors (*β* = 26.94, *p* = 0.02), whereas no such relationship was present when the same participants were under placebo (*β* = 9.59, *p* = 0.40). Next, we examined the relationship between the stimulus stickiness parameter from the computational model and the raw data measure of perseveration. Stimulus stickiness during reversal was not significantly correlated with the raw data measure of perseveration, in either the placebo (*β* = 4.13, *p* = 0.50) or LSD (*β* = 11.60, *p* = 0.09) condition. Further exploratory analyses are reported in the online Supplementary material.

## Discussion

There has been a recent surge of interest in the potential therapeutic effects of psychedelics, including LSD. Theorising on the mechanisms of such effects centres on their role in enhancing learning and plasticity. In the current study, we tested these postulated effects of LSD in flexible learning in humans and find that LSD increased learning rates, exploratory behaviour, and the impact of previously learnt values on subsequent perseverative behaviour. Specifically, LSD increased the speed at which value representations were updated following prediction error (the mismatch between expectations and experience). Whilst LSD enhanced the impact of both positive and negative feedback, overall it augmented learning from reward significantly more than it augmented learning from punishment.

The observation that LSD enhanced learning rates may be particularly important for understanding the mechanisms through which LSD might be therapeutically useful. Psychedelic drugs have been hypothesised to destabilise pre-existing beliefs (relax prior beliefs or ‘priors’), making them amenable to revision (Carhart-Harris & Friston, 2019). The notion of relaxed priors is directly compatible with increased RL rates: in our study, LSD rendered subjects more sensitive to prediction errors, which naturally implies downweighting of prior beliefs (Carhart-Harris & Friston, 2019). That LSD affected a fundamental belief-updating process is notable given that psychedelics are under investigation trans-diagnostically for diverse clinical disorders including depression (Carhart-Harris et al., 2016a, 2018, 2021; Goldberg et al. 2020; Ross et al., 2016), anxiety (Goldberg et al. 2020; Griffiths et al., 2016; Grob et al., 2011), alcohol (Bogenschutz et al., 2015) and nicotine abuse (Johnson, Garcia-Romeu, Cosimano, & Griffiths, 2014), obsessive–compulsive disorder (OCD) (Moreno, Wiegand, Taitano, & Delgado, 2006), and eating disorders (Lafrance et al., 2017). A unifying feature of these conditions is intransigent maladaptive associations in need of revision.

Behaviour was more exploratory overall under LSD, as assessed computationally in two ways, consistent with theoretical accounts of psychedelic effects which have predicted increased exploratory tendencies (Carhart-Harris & Friston, 2019). First, LSD decreased stimulus stickiness, which indicates a diminished tendency to repeat previously chosen options, irrespective of reinforcement history (value-free). This effect on stickiness was significant in all phases of the experiment – when considering the entire experiment as a whole (acquisition and reversal), when examining initial learning only (acquisition), and when isolating the reversal phase. In other words, regardless of LSD-induced changes in value-guided choice strategies (elaborated upon below), LSD promoted an overall latent tendency to explore in the form of shifting between choices, irrespective of feedback and value, which was maintained during both stable and changing circumstances. That LSD lowered stimulus stickiness may also be clinically relevant: stimulus stickiness was recently shown to be abnormally high in cocaine and amphetamine use disorders (Kanen et al., 2019).

LSD also modulated value-based exploratory tendencies (indexed by the reinforcement sensitivity parameter), which, by contrast, differed by phase. When looking at the experiment as a whole, there was no effect of LSD on reinforcement sensitivity, although lack of an effect here was obscured by the following patterns: When examining initial learning only, reinforcement sensitivity was substantially diminished under LSD, indicating a tendency for increased exploration away from the more highly valued choice option. During the reversal phase, meanwhile, reinforcement sensitivity was increased, indicative of a heightened tendency to exploit the choice option that was computed to be more highly valued trial-by-trial, which can be seen as adaptive when circumstances change, and rapid reorienting of actions is required.

A shift in the computations underlying choice was also observed in relation to RL rates, during learning to maximise reward and minimise punishment in an initial situation and when adapting actions following contingency reversal. Whereas overall, LSD enhanced both the reward and punishment rates (especially for rewards), the increase in punishment learning rate appeared during the reversal phase only. The reward learning rate was elevated in both the acquisition and reversal phases. Together, these learning rate findings suggest that LSD accelerates the updating of value, in a way that is (overall) especially reward-driven, and LSD speeds up learning from negative feedback that is encountered when circumstances change.

Under LSD, better initial learning led to more perseverative responding. The implication is that when a behaviour is newly and more strongly learned through positive reinforcement (i.e. the acquisition phase) under LSD, it may persist more strongly even when that action is no longer relevant (i.e. the reversal phase). These measures of overt performance defined based on feedback are orthogonal to an overall latent tendency towards exploration irrespective of reinforcement history (low stimulus stickiness). Importantly, perseveration (den Ouden et al., 2013) itself, as assessed in the analysis of raw data measures, was not elevated by LSD, nor did it correlate with stimulus stickiness (online Supplementary Table S3).

Given the broad effect of LSD on a range of neurotransmitter systems (Nichols, 2004, 2016), it is not possible to determine the specific neurochemical mechanism underlying the observed LSD effects on learning. Nonetheless, obvious possibilities involve the serotonin and dopamine systems, in particular 5-HT_2A_ and D_2_ receptors (Marona-Lewicka et al., 2005; Marona-Lewicka & Nichols, 2007; Nichols, 2004, 2016). Specifically, the psychological plasticity purportedly promoted by psychedelics is believed to be mediated through action at 5-HT_2A_ receptors (Carhart-Harris & Nutt, 2017) via downstream enhancement of glutamatergic activity (Barre et al., 2016) and brain-derived neurotrophic factor (BDNF) expression (Hutten et al., 2021; Vaidya et al., 1997). The hypothesis that the present results regarding RL rates are driven by the serotonergic effects of LSD is supported by two recent studies in mice. Optogenetically stimulating dorsal raphé serotonin neurons enhanced RL rates (Iigaya, Fonseca, Murakami, Mainen, & Dayan, 2018), whilst activation of these neurons tracked both reward and punishment prediction errors during reversal learning (Matias et al., 2017). Neurotoxic manipulation of serotonin in marmoset monkeys during PRL, meanwhile, altered stimulus stickiness (Rygula et al., 2015): this implicates a serotonergic mechanism underlying increased exploratory behaviour following LSD administration in the present study.

In addition to affecting the serotonin system, however, LSD also acts at dopamine receptors (Nichols, 2004, 2016), albeit with a far lower direct affinity for dopamine receptors than for 5-HT receptors. Dopamine has long been known to play a crucial role in belief updating following reward (Schultz et al., 1997), and more recent evidence shows that dopaminergic manipulations may alter learning rates (Kanen et al., 2019; Schultz, 2019; Swart et al., 2017). A dopaminergic effect would be in line with our previous study where genetic variation in the dopamine, but not serotonin transporter polymorphism, was associated with the same enhanced relationship between acquisition and perseveration as reported here under LSD (den Ouden et al., 2013).

Serotonin–dopamine interactions represent another candidate mechanism that could underlie the present findings. For example, stimulation of 5-HT_2A_ receptors in the prefrontal cortex of the rat enhanced ventral tegmental area dopaminergic activity (Bortolozzi, Díaz-Mataix, Scorza, Celada, & Artigas, 2005). Indeed, the initial action of LSD at 5-HT_2A_ receptors has been proposed to sensitise dopamine neuron firing (Nichols, 2016). LSD action at D_2_ receptors, albeit with a low binding affinity, may be more pronounced in a late phase of LSD's effects (Marona-Lewicka et al., 2005; Marona-Lewicka & Nichols, 2007), which may be relevant given the relatively long delay between LSD administration and performance of the current task (see Methods). However, arguing against a late dopaminergic effect is a previous study in rodents where the effects of LSD on reversal learning were consistent across four different time lags between drug administration and behavioural testing (King, Martin, & Melville, 1974).

The result of the enhanced coupling of acquisition learning and perseverative responding under LSD is in line with a recent study showing that LSD induced higher-order cognitive inflexibility in a set-shifting paradigm (Pokorny et al., 2019). Importantly, these effects were blocked by co-administration of the 5-HT_2A_ antagonist ketanserin (Pokorny et al., 2019), showing that the LSD-induced impairments were mediated by 5-HT_2A_ agonism, consistent with a 5-HT_2A_ mechanism underlying the present results.

LSD's effects to increase acquisition-perseveration coupling and worsen set-shifting (Pokorny et al., 2019), in conjunction, suggest that what is newly or recently learnt through reinforcement under LSD is more ‘stamped in’, and thus may subsequently be harder to update. Whilst these findings are ostensibly at odds with the observation that LSD enhanced plasticity (through enhanced learning rates), they can be reconciled by considering the timing of drug administration with respect to initial learning and tests of cognitive flexibility. In both the present experiment and the previous set-shifting study (Pokorny et al., 2019), all phases of learning (acquisition and reversal) were conducted after LSD administration. In contrast, when acquisition learning was conducted prior to LSD administration, LSD resulted in improved reversal learning (using a reversal paradigm in rats; King et al., 1974). Likewise, when acquisition learning was conducted prior to the administration of a 5-HT_2A_ antagonist, reversal learning was impaired (Boulougouris et al., 2008; also see Furr et al., 2012). Collectively, these findings suggest that whether a prior belief is down- or up-weighted under LSD may depend on whether the prior is formed before or during drug administration, respectively. This observation is of great relevance for a putative therapeutic setting, where maladaptive beliefs will have been formed before treatment.

Another important consideration for reconciling the effects of 5-HT_2A_ receptor modulation on behavioural/cognitive flexibility is that 5-HT_2A_ antagonism can produce opposite effects depending on whether the OFC or striatum is targeted (Amodeo et al., 2017), complicating the interpretation of studies employing systemic administration (Amodeo et al., 2014, 2020; Baker et al., 2011; Odland et al., 2021). Species, strain, dose, compound, route of administration, task specifications (and engagement of cortical and subcortical structures), and reinforcement schedule must also be considered. The application of computational modelling may also help unify effects across studies and species.

While we observed an effect of LSD on acquisition-perseveration coupling, reminiscent of a previous similar observation as a function of genetic variability in the dopamine transporter (den Ouden et al., 2013), we did not observe effects of LSD on acquisition performance or perseveration directly, or on lose-stay and win-stay behaviour, unexpectedly. In fact, more broadly, the effects of LSD observed here differ from the effects of neurochemically more specific influences such as acute serotonin reuptake inhibition (Bari et al., 2010; Skandali et al., 2018), or neurotoxic serotonin depletion (Bari et al., 2010; Rygula et al., 2015). More in line with this, previous studies with LSD administration, examining perseveration, using an outcome devaluation paradigm, found no effect of LSD (Hutten et al., 2020), nor did a study on visual memory during paired associates learning (Family et al., 2020).

Our computational modelling approach, here, was more sensitive to detecting the effects of LSD. It may be possible to reconcile these robust computational effects with the minimal overt behavioural performance effects via the following speculation. Subtle differences in states of underlying plasticity may not translate to overt differences in instrumental or Pavlovian responses, even if the long-term expression of these learned responses would differ. For example, in the memory reconsolidation literature, a previously learned associative memory is believed to become susceptible to disruption (e.g. pharmacologically or behaviourally) following cued reactivation or recall for a period of several hours known as the ‘reconsolidation window’ (Lee, Nader, & Schiller, 2017). There is evidence that conducting extinction training (learning) during the reconsolidation window – when mechanisms of plasticity differ – does not alter the overt success or failure of extinction within the session, yet there are long-term effects; extinction learning during the reconsolidation window can be more enduring than extinction learned outside of this window (Schiller, Kanen, LeDoux, Monfils, & Phelps, 2013; Steinfurth et al., 2014). These Pavlovian extinction learning data, showing no difference during extinction itself, may parallel the instrumental conditioning data in the present study, in that we report no observable effect of LSD on most raw data measures (e.g. number of correct responses), yet latent learning processes that relate to purported mechanisms of plasticity, namely learning rate, were affected. Future studies would need to determine whether and how to harness this apparent window of heightened plasticity for therapeutic benefit.

Limitations of this study include the following. We have made a case for the critical involvement of the 5-HT_2A_ receptor; however, we cannot be sure which particular receptor interaction(s) the current findings are caused by. LSD, in addition to binding with high affinity to 5-HT_2A_ receptors, acts at numerous other receptors including D_1_, D_2_, 5-HT_1A/1B/1D_, 5-HT_2C_, 5-HT_5A_, 5-HT_6_, and 5-HT_7_ (Nichols, 2004). Indeed, 5-HT_2C_ receptors can counter 5-HT_2A_ effects on reversal learning (Boulougouris et al., 2008). A future study co-administering LSD with a 5-HT_2A_ antagonist would help discern the putative 5-HT_2A_-mediated effects. Additionally, the subjective effects and plasma levels of LSD were not measured at the time of task administration. Furthermore, even though our parameter recovery analysis was successful (see online Supplementary material), we were unable to demonstrate the initial learning-perseveration effect observed in the behavioural data in the simulated data.

In summary, the core result of this study was that LSD enhanced the rate at which humans updated their beliefs based on feedback. RL was most enhanced by LSD when receiving the reward, and to a lesser extent following punishment. LSD also increased exploratory behaviour. These findings have implications for understanding the mechanisms through which LSD might be therapeutically useful for revising deleterious associations.

## Supplementary material

For supplementary material accompanying this paper visit https://doi.org/10.1017/S0033291722002963. click here to view supplementary material
