# Supplementary material for: Effect of lysergic acid diethylamide (LSD) on reinforcement learning in humans
Source: Psychol Med. 2022 Nov 22;53(14):6434–45. doi: 10.1017/S0033291722002963 (PMC10600934; doi:10.1017/S0033291722002963)
Supplement: Supplementary file 1 [file S0033291722002963sup001.docx]

**Effect of lysergic acid diethylamide (LSD) on reinforcement learning in humans**

Jonathan W. Kanen^#^*^1,2^, Qiang Luo*^2,3,4,5^, Mojtaba Rostami Kandroodi^6,7^, Rudolf N. Cardinal^2,8,9^, Trevor W. Robbins^1,2^, David J. Nutt^10^, Robin L. Carhart-Harris^11^, Hanneke E.M. den Ouden^7^

^1^Department of Psychology, University of Cambridge, Cambridge, UK

^2^Behavioural and Clinical Neuroscience Institute, University of Cambridge, Cambridge, UK

^3^National Clinical Research Center for Aging and Medicine at Huashan Hospital, State Key Laboratory of Medical Neurobiology and Ministry of Education Frontiers Center for Brain Science, Institutes of Brain Science and Institute of Science and Technology for Brain-Inspired Intelligence, Fudan University, Shanghai, 200433, China

^4^Center for Computational Psychiatry, Ministry of Education-Key Laboratory of Computational Neuroscience and Brain-Inspired Intelligence, Human Phenome Institute, Fudan University, Shanghai, 200032, China

^5^Shanghai Key Laboratory of Mental Health and Psychological Crisis Intervention, School of Psychology and Cognitive Science, East China Normal University, Shanghai, 200241, China

^6^Department of Cognitive Science and Artificial Intelligence, Tilburg University, Tilburg, The Netherlands

^7^Donders Institute for Brain, Cognition and Behaviour, Radboud University, Nijmegen, The Netherlands

^8^Department of Psychiatry, University of Cambridge, Cambridge, UK

^9^Cambridgeshire and Peterborough NHS Foundation Trust, UK

^10^Centre for Psychedelic Research, Department of Brain Sciences, Imperial College London, United Kingdom

^11^Neuroscape Psychedelics Division, University of California San Francisco, San Francisco, California, USA

**^#^Corresponding author:** Jonathan W. Kanen, email: [jonathan.kanen@gmail.com](mailto:jonathan.kanen@gmail.com)

*Joint first authorship

**Supplementary information**

**Subjective effects of LSD**

The subjective effects of LSD were assessed by visual analogue scale-style (VAS) ratings and the 11-factor altered states of consciousness (ASC) questionnaire (Studerus et al., 2010). VAS items that were rated significantly higher on LSD than on placebo, following Bonferonni correction, included “my imagination was extremely vivid,” “the experience had a dream-like quality,” “sounds influenced things I saw,” “my sense of size and space was distorted,” “I felt unusual bodily sensations,” “my thoughts wandered freely,” “my perception of time was distorted,” “I saw geometric patterns,” “edges appeared warped,” “my thinking was muddled,” “I saw movement in things that weren’t really moving,” “I experienced a sense of merging with my surroundings,” “things looked strange,” and “I felt like I was floating.” Participants rated a control item “I felt entirely normal” higher on placebo than on LSD. All factors on the ASC questionnaire were increased after Bonferonni correction, aside from the anxiety factor, which included: complex imagery, elementary imagery, audio/visual synaesthesia, meaning, experience of unity, spiritual experience, blissful state, insightfulness, disembodiment, and impaired cognition. Positive mood, furthermore, was significantly increased under LSD as well (also assessed by a VAS). Further details on the subjective effects of LSD in this sample of participants are published in Carhart-Harris et al. (2016).

**Relationship between subjective effects and behavioural measures**

We conducted exploratory correlations between subjective effects and the core behavioral measures of the paper – reward learning rate, punishment learning rate, stimulus stickiness, reinforcement sensitivity, correct responses during acquisition, perseverative responses during reversal. Results are displayed in Supplementary Tables 4 and 5.

**Simulation: Methods**

We simulated behavioural data from the winning model to determine how behavioural patterns in the synthetic data compared to the raw data. Simulated data were analysed for win-stay probability, lose-stay probability, acquisition performance, and perseveration, as was done for the original raw data analysis. For each condition (placebo and LSD), we simulated 100 “virtual subjects” using the posterior mean parameters from that condition, from the winning model, per Kanen et al. (2019).

**Simulation: Results**

Simulated behavioural data, generated using parameter estimates from the winning model, were analysed using summary statistics in order to assess whether the winning model could capture the observed effects of LSD on raw behaviour. Simulated data are shown in Supplementary Figure 1. Consistent with the original data, lose-stay probability was unaffected by LSD in the simulated behaviour (*t*_99_ = –0.37, p = .71, d = .03) and acquisition performance was also unaffected (*t*_99_ = 0.25, p = .81, d = .03). Perseveration was enhanced by LSD in the simulation (*t*_99_ = –2.24, *p* = 0.03, *d* = .22), which differs slightly from, yet is in line with, the original analyses showing an enhanced relationship between acquisition and perseveration under LSD. Linear regression examining whether correct responses during the acquisition phase (LSD minus placebo) predicted more perseverative errors in the reversal stage (LSD minus placebo) was not significant in the simulated data (*β* = 0.15, *p* = 0.13). Separate regressions for each condition also showed no significant relationship between acquisition performance and perseveration for LSD (*β* = 0.12, *p* = 0.25) or for placebo (*β* = 0.01, *p* = .91). Win-stay probability was diminished under LSD in the simulated data (*t*_99_ = 11.91, *p* = 8.21 × 10^–21^, *d* = 1.19) whereas it was unaffected by LSD in the raw data analysis.

**Parameter recovery**

Similar to our previous publication that employed the same modelling approach (Kanen et al. 2019), we performed simulations (as above) of the winning model using the parameter values recovered from the experimental data. Applying our models to these simulated behavioural data, we tested whether the model can recover the true parameter values from the simulation. If a true parameter value falls within the HDI of the fitted parameter in our model, then we consider this a successful parameter recovery.  This simulation was repeated 30 times, and the success rates of parameter recovery are reported below. The winning model was Model 3 with the following parameter values: reward learning rate = 0.2, punishment learning rate = 0.4, reinforcement sensitivity = 5.5, and stimulus stickiness = 0.4. We found that among these 30 simulations, the reward learning rate was successfully recovered 28 times (93%), the punishment learning rate was recovered 29 times (97%), reinforcement sensitivity was recovered 26 times (87%), and stimulus stickiness was recovered 30 times (100%). The mean squared errors of these estimations were 0.0001, 0.0002, 0.0446, and 0.0006 for the reward learning rate, punishment learning rate, reinforcement sensitivity, and stimulus stickiness, respectively.

**Supplementary Figure 1.** Simulated data. **A)** Trial-by-trial average probability of choosing each stimulus, averaged over simulated subjects, separated by drug session. A sliding 5-trial window was used for smoothing. The vertical dotted line indicates the reversal of contingencies. Shading indicates ±1 standard error of the mean (SE). **B)** Relationship between initial learning and perseveration on LSD versus placebo in simulated data. Shading indicates ±1 SE. **C)** Distributions depicting the average per-subject probability (scattered dots) of simulated subjects choosing each stimulus while under placebo (shown in dark blue) and LSD (light blue). The mean value for each distribution is illustrated with a single dot at the base of each distribution, and the mean values for the probability of choosing different stimuli in each condition are connected by a line. Black error bars around the mean value show ±1 SE. The horizontal dotted line indicates chance-level “stay” behaviour (33%). **D)** Distributions depicting the average per-subject probability (scattered dots) of simulated subjects repeating a choice (staying) after receiving positive or negative feedback under placebo (dark blue) and LSD (light blue). The horizontal dotted line indicates chance-level “stay” behaviour (33%).


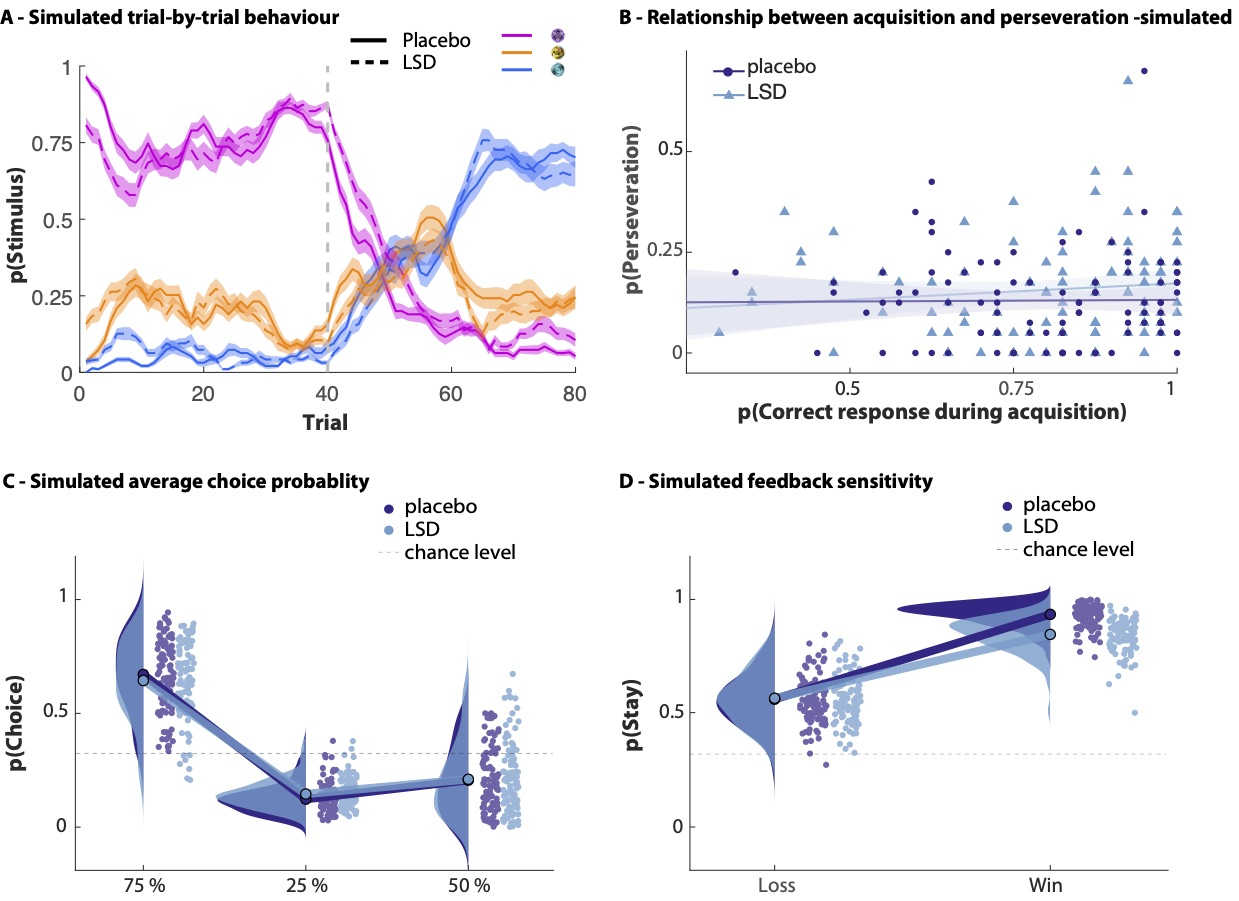


**Supplementary Figure 2.**

Results from Model 1, which did not win in the model comparison procedure. All trials (acquisition and reversal) were included in this analysis. Contrasts with the posterior 95% (or greater) highest posterior density interval [HDI] of the difference between means excluding zero (0 ∉ 95% HDI) are shown in red. The reward and punishment learning rates were significantly increased, the reinforcement sensitivity was not modulated, and stimulus stickiness was not included in this model. These results resemble the winning model presented in the main text, save for the omission of the stimulus stickiness parameter here.

**Supplementary Figure 3.**

Results from Model 2, which did not win in the model comparison procedure. All trials (acquisition and reversal) were included in this analysis. Contrasts with the posterior 95% (or greater) highest posterior density interval [HDI] of the difference between means excluding zero (0 ∉ 95% HDI) are shown in red. The single learning rate was decreased, the reinforcement sensitivity was increased, and stimulus stickiness was unaffected. These data highlight the importance (in the present study) of having two separate learning rates for rewarding and punishing outcomes.

**Supplementary Table 1**

Demographic information.

| Sex (M:F) | 15:4 |
| --- | --- |
| Age | 30.6 (mean); 7.8 (SD); 22 (min); 47 (max) |
| BMI (n = 13) | 22.4 (mean); 1.6 (SD); 20 (min); 26.1 (max) |
| Alcohol: units per week (n = 18) | 10 (mean); 9 (SD); 0 (min); 28 (max) |
| Cannabis: times used (n = 16) | 606 (mean); 547 (SD); 30 (min); 2000 (max) |
| Cigarette smoking | 17 of 19 were non-smokers |

**Supplementary Table 2**

Mean and standard error of the number of correct responses and errors before and after reversal, and the number of perseverative errors. PLA = placebo.

|  | PLA | | LSD | |
| --- | --- | --- | --- | --- |
|  | Mean | 1 standard error | Mean | 1 standard error |
| Correct responses (acquisition) | 27.26 | 1.976 | 25.47 | 2.092 |
| Correct responses (reversal) | 18.16 | 1.867 | 17.63 | 1.889 |
| Incorrect responses (acquisition) | 12.74 | 1.976 | 14.53 | 2.092 |
| Incorrect responses (reversal) | 21.84 | 1.867 | 22.37 | 1.889 |
| Perseveration | 8.74 | 1.361 | 8.68 | 1.560 |

**Supplementary Table 3**

Summary of correlations between raw data behavioural measures and parameters from the computational model. *p* values are displayed. No significant correlations survived correction for 192 comparisons, using the Benjamini-Hochberg method at q = .15 (Skandali et al. 2018). Orange shading indicates positive correlation; blue shading indicates negative correlation. *α^rew^*= reward learning rate, *α^pun^*= punishment learning rate, *τ^reinf^*= reinforcement sensitivity, *τ^stim^* = stimulus stickiness.

|  | **Acquisition Performance**  **(errors)** | | **Perseveration**  **(consecutive errors)** | | **Lose-Stay*^all^*** | | **Win-stay*^all^*** | | **Lose-Stay*^acq^*** | | **Win-stay*^acq^*** | | **Lose-Stay*^rev^*** | | **Win-Stay*^rev^*** | | |
| --- | --- | --- | --- | --- | --- | --- | --- | --- | --- | --- | --- | --- | --- | --- | --- | --- | --- |
|  | **PLA** | **LSD** | **PLA** | **LSD** | **PLA** | **LSD** | **PLA** | **LSD** | **PLA** | **LSD** | **PLA** | **LSD** | **PLA** | **LSD** | **PLA** | **LSD** |  |
| ***α^rew,all^*** | .9 | .053 | .9 | .015 | .79 | .37 | .55 | .11 | .9 | .3 | .6 | .1 | .7 | .4 | .5 | .2 |  |
| ***α^pun,all^*** | .18 | .053 | .3 | .6 | .01 | .04 | .08 | .65 | .005 | .017 | .09 | .7 | .036 | .17 | .09 | .7 |  |
| ***τ^reinf,all^*** | 5.9×10^-4^ | .003 | .3 | .024 | 1×10^-3^ | .01 | .01 | .02 | 2.25×10^-4^ | .015 | .016 | .026 | .005 | .043 | .01 | .025 |  |
| ***τ^stim,all^*** | .024 | .162 | .4 | .13 | 2×10^-3^ | .03 | .07 | .02 | .011 | .013 | .036 | .033 | 6.28×10^-4^ | .094 | .18 | .03 |  |
| ***α^rew,acq^*** | .8 | 1×10^-4^ | .4 | .017 | .3 | .13 | .8 | .049 | .3 | .14 | .8 | .007 | .3 | .16 | .8 | .3 |  |
| ***α^pun,acq^*** | .024 | .01 | .3 | .2 | .001 | .007 | .23 | .5 | 8.09 × 10^-4^ | .002 | .23 | .6 | .005 | .068 | .24 | .5 |  |
| ***τ^reinf,acq^*** | 8×10^-6^ | .009 | .19 | .006 | 1.58×10^-4^ | .021 | 9.42×10^-4^ | .011 | 6.2×10^-5^ | .008 | 8.07×10^-4^ | .013 | .001 | .1 | .003 | .024 |  |
| ***τ^stim,acq^*** | .07 | .4 | .5 | .21 | .03 | .12 | .051 | .005 | .053 | .057 | .009 | .009 | .024 | .3 | .21 | .02 |  |
| ***α^rew,rev^*** | 1 | .8 | .09 | .9 | .9 | .4 | .17 | .3 | .8 | .6 | .3 | .6 | 1 | .4 | .11 | .22 |  |
| ***α^pun,rev^*** | .035 | .084 | .4 | .5 | 7.21×10^-4^ | .017 | .5 | 1 | .004 | .029 | .5 | 1 | 3.66×10^-4^ | .034 | .5 | 1 |  |
| ***τ^reinf,rev^*** | 8.54×10^-4^ | .016 | .5 | 6×10^-4^ | .009 | .116 | .001 | 5.83×10^-4^ | .005 | .09 | .004 | .005 | .024 | .21 | .001 | 6.47×10^-4^ |  |
| ***τ^stim,rev^*** | .024 | .036 | .5 | .09 | 5.32×10^-4^ | 7.76×10^-4^ | .048 | .005 | .006 | .002 | .051 | .026 | 7.6×10^-5^ | .002 | .08 | .006 |  |

**Supplementary Table 4**

P-values for exploratory correlations between subjective effects of LSD and raw data behavioural measures from the probabilistic reversal learning task. No significant correlations survived correction for 96 comparisons, using the Benjamini-Hochberg method at q = .15 (Skandali et al. 2018). All correlation coefficients were negative. Shading relevant only when there was an uncorrected significant result. Orange shading indicates positive correlation (not present). Blue shading indicates negative correlation (not significant). PLA = placebo.

|  | **Win-Stay** | | **Lose-Stay** | | **Acquisition performance**  **(errors)** | | **Perseveration**  **(consecutive errors)** | |
| --- | --- | --- | --- | --- | --- | --- | --- | --- |
|  | **PLA** | **LSD** | **PLA** | **LSD** | **PLA** | **LSD** | **PLA** | **LSD** |
| **Positive mood** | .7 | .8 | .8 | .4 | 1.0 | .4 | .7 | 1.0 |
| **Experience of unity** | .8 | .5 | .7 | .2 | .5 | .07 | .5 | .048 |
| **Spiritual experience** | .8 | .5 | .6 | .4 | .5 | .050 | .5 | .040 |
| **Blissful state** | .9 | .7 | .6 | .3 | .6 | .11 | .4 | .10 |
| **Insightfulness** | .8 | .8 | .6 | .23 | .6 | .3 | .3 | .3 |
| **Disembodiment** | .22 | .3 | .9 | 1.0 | .8 | .6 | .14 | .8 |
| **Impaired cognition** | .7 | .4 | .5 | .4 | .5 | .4 | .9 | .5 |
| **Anxiety** | .8 | .6 | .6 | .5 | .6 | .3 | .4 | .5 |
| **Complex imagery** | .8 | .5 | .7 | .6 | .6 | .11 | .3 | .13 |
| **Elementary imagery** | .8 | .5 | .7 | .2 | .5 | .3 | .3 | 1.0 |
| **Audio/visual synaesthesia** | .9 | .4 | .7 | .2 | .5 | .3 | .4 | .039 |
| **Meaning** | .8 | 1.0 | .5 | .056 | .3 | .029 | .4 | .5 |

**Supplementary Table 5**

P-values for exploratory correlations between subjective effects of LSD and behavioural measures from the probabilistic reversal learning task, derived from the computational model. Parameter estimates from the computational model are presented for all trials (acquisition and reversal). No significant correlations survived correction for 96 comparisons, using the Benjamini-Hochberg method at q = .15 (Skandali). Shading relevant only when there was an uncorrected significant result. Orange shading indicates positive correlation (not present). Blue shading indicates negative correlation (not significant).

|  | **Reward**  **Learning**  **Rate, *α^rew^*** | | **Punishment**  **Learning**  **Rate, *α^pun^*** | | **Reinforcement**  **Sensitivity,**  ***τ^reinf^*** | | **Stimulus**  **Stickiness,**  ***τ^stim^*** | |
| --- | --- | --- | --- | --- | --- | --- | --- | --- |
|  | **PLA** | **LSD** | **PLA** | **LSD** | **PLA** | **LSD** | **PLA** | **LSD** |
| **Positive mood** | .7 | .3 | .9 | .5 | .5 | .5 | .3 | .3 |
| **Experience of unity** | .12 | .6 | .3 | .23 | .5 | .003 | .5 | .6 |
| **Spiritual experience** | .14 | .11 | .22 | .12 | .4 | .07 | .6 | .5 |
| **Blissful state** | .11 | .5 | .3 | .22 | .4 | .010 | .5 | .3 |
| **Insightfulness** | .12 | .4 | .23 | .24 | .3 | .028 | .6 | .14 |
| **Disembodiment** | .9 | .4 | .6 | .9 | .4 | .09 | .5 | .16 |
| **Impaired cognition** | .040 | .6 | .14 | .5 | .7 | .9 | .7 | .16 |
| **Anxiety** | .12 | .5 | .15 | .5 | .3 | .7 | .6 | .25 |
| **Complex imagery** | .3 | .11 | .35 | .5 | .3 | .022 | .4 | .23 |
| **Elementary imagery** | .3 | .4 | .5 | .9 | .3 | .9 | .4 | .12 |
| **Audio/visual synaesthesia** | .17 | .032 | .4 | .9 | .3 | .08 | .4 | .9 |
| **Meaning** | .9 | .11 | .25 | .3 | .4 | .053 | .9 | .3 |

**Supplementary Table 6**

Summary of exploratory correlations between raw data measures from the probabilistic reversal learning task within and across the placebo and LSD conditions. Orange: positive correlation, blue: negative correlation (not present). Significant correlations survived correction for 28 comparisons, using the Benjamini-Hochberg method at q = .15 (Skandali et al. 2018).

|  |  | **Win-Stay** | | **Lose-Stay** | | **Acquisition**  **Performance**  **(errors)** | | **Perseveration**  **(consecutive errors)** | |
| --- | --- | --- | --- | --- | --- | --- | --- | --- | --- |
|  |  | **PLA** | **LSD** | **PLA** | **LSD** | **PLA** | **LSD** | **PLA** | **LSD** |
| **Win-Stay** | **PLA** |  | .7 | .13 | .7 | .033 | .3 | .12 | .049 |
|  | **LSD** |  |  | .016 | .039 | .3 | .004 | .4 | .001 |
| **Lose-Stay** | **PLA** |  |  |  | 8.12×10^-4^ | 7×10^-6^ | .001 | .6 | .007 |
|  | **LSD** |  |  |  |  | .032 | 5x10^-4^ | .9 | .036 |
| **Acquisition**  **Performance**  **(errors)** | **PLA** |  |  |  |  |  | .050 | .8 | .003 |
|  | **LSD** |  |  |  |  |  |  | .5 | .003 |
| **Perseveration**  **(consecutive errors)** | **PLA** |  |  |  |  |  |  |  | .6 |
|  | **LSD** |  |  |  |  |  |  |  |  |

**Supplementary Table 7**

Relationship between alcohol use, cannabis use, and age, with the core learning measures. There were no significant correlations. *p* values displayed. PLA = placebo.

|  |  | Age | Alcohol: units per week (n = 18) | Cannabis: times used (n = 16) |
| --- | --- | --- | --- | --- |
| **Win-Stay** | PLA | .9 | .9 | 1.0 |
|  | LSD | .2 | .4 | 1.0 |
| **Lose-Stay** | PLA | .7 | .8 | .9 |
|  | LSD | .9 | .9 | .3 |
| **Acquisition**  **Performance**  **(errors)** | PLA | .5 | .8 | .8 |
|  | LSD | .3 | .9 | .9 |
| **Perseveration**  **(consecutive errors)** | PLA | .8 | 1.0 | .6 |
|  | LSD | 1.0 | .7 | .7 |
| ***α^rew,all^*** | PLA | .5 | .14 | .4 |
|  | LSD | .3 | .2 | .8 |
| ***α^pun,all^*** | PLA | .8 | .8 | .8 |
|  | LSD | .9 | .7 | .7 |
| ***τ^reinf,all^*** | PLA | .8 | .7 | 1.0 |
|  | LSD | .8 | .7 | 1.0 |
| ***τ^stim,all^*** | PLA | .7 | .4 | .9 |
|  | LSD | .7 | .4 | .9 |

**Additional references**

Studerus E, Gamma A, Vollenweider FX (2010) Psychometric evaluation of the altered states of consciousness rating scale (OAV). PLoS One 5. doi: 10.1371/journal.pone.0012412
